# A multicenter observational safety study in Swedish children and adolescents using insulin detemir for the treatment of type 1 diabetes

**DOI:** 10.1111/pedi.12019

**Published:** 2013-03-01

**Authors:** Annelie Carlsson, Gun Forsander, Johnny Ludvigsson, Sara Larsen, Eva Örtqvist, on behalf of the Swedish PREDICTIVE-Youth Study Group

**Affiliations:** a Department of Clinical Sciences, SUS University Hospital, Lund UniversityLund, Sweden; b Department of Pediatrics, The Queen Silvia Children’s Hospital, Sahlgrenska University HospitalGöteborg, Sweden; c Divison of Pediatrics, Department of Clinical and Experimental Medicine, Linköping University and Östergötland County CouncilLinköping, Sweden; d Novo Nordisk Scandinavia ABMalmö, Sweden; e Division of Pediatrics, Astrid Lindgrens Children’s Hospital, Karolinska University HospitalStockholm, Sweden

**Keywords:** detemir, pediatrics, safety, type 1 diabetes

## Abstract

This 26-wk observational study in children and adolescents with type 1 diabetes (T1D) in Sweden investigated the safety and efficacy of insulin detemir (IDet) in newly diagnosed (ND) patients and those with established diabetes (ED) switching to IDet. A total of 159 patients initiated IDet as part of basal–bolus therapy, 59 in the ND stratum (mean age 9.7 yr) and 97 in the ED stratum (mean age 12.5 yr). The primary outcome measure was the incidence of severe adverse drug reactions; just one major hypoglycemic event occurred in a patient in the ND stratum during the study and one patient was withdrawn due to injection-site reactions. All other events were classified as mild. In the ED stratum, there was a reduction in hypoglycemic events in the 4 wk prior to study end from baseline (mean reduction of 2.46 events, not significant) and a significant reduction in nocturnal hypoglycemia (mean reduction of 2.24 events, p = 0.0078). Glycemic control improved in the ND stratum as expected and, in the ED stratum, there was no significant change in HbA1c from baseline (mean reduction of −0.45%). At study end, mean daily IDet doses were 0.39 U/kg (ND) and 0.54 U/kg (ED). Weight increased by 5.7 and 2.0 kg in the ND and ED strata, respectively, and was within the normal limits for growing children. IDet provided good glycemic control and was well tolerated, with a reduced risk of nocturnal hypoglycemia in a heterogeneous cohort of children and adolescents with T1D.

Basal–bolus insulin therapy is the gold-standard treatment in patients with type 1 diabetes (T1D). Insulin is required to achieve glycemic control and to delay the onset of diabetic complications resulting from prolonged hyperglycemia 1. Patients with intensive insulin treatment reaching lower glycemic targets had a reduced risk of retinopathy and nephropathy 2–3, and decreasing HbA1c has also been associated with reduced diabetic complications in nonselected patient populations 4–5. However, intensive insulin therapy may be associated with an increase in adverse effects, particularly hypoglycemia and weight gain 2–6. Hypoglycemia is more common in children than adults, and often requires third-party assistance 3–7. The consequences of hypoglycemia are more pronounced in the pediatric population compared with adults, and may have severe consequences, as reviewed by Bjørgaas 8. Several studies have suggested that severe hypoglycemia may result in long-term brain abnormalities in children, such as excitotoxic damage, seizures, and cognitive decline 8–11; as a result, physicians may be reluctant to increase insulin dose to reach optimal HbA1c targets 12. A patient’s fear of hypoglycemia, and especially a parent’s fear of hypoglycemia in their child, can also lead to reduced treatment adherence 13.

Several other factors lead to challenges in achieving glycemic control in children. Firstly, younger children are largely dependent on carers to administer their insulin. Secondly, children and adolescents have changing routines and varied eating patterns, which can make adhering to insulin treatment regimens cumbersome. Thirdly, weight gain can be problematic with insulin therapy, particularly in adolescent females, who are prone to adiposity 14. Fourthly, psychosocial problems, such as broken homes and difficulties in understanding the complicated treatment regimens, may contribute to a lack of adherence.

In Sweden, the majority of patients with T1D follow a basal–bolus insulin regimen, whereby a long-acting basal insulin is injected once or twice daily and a fast-acting bolus insulin is injected prior to meals, with up to 40% of Swedish pediatric patients using insulin pumps for administration 15. An ideal basal insulin for children and adolescents would provide a physiological, flexible, and predictable profile, with a low risk of hypoglycemia. Insulin detemir (IDet) is a long-acting basal insulin analog that has been tested in many patients as part of a basal–bolus regimen in randomized, controlled clinical trials and demonstrated a beneficial safety:efficacy ratio, with more predictable glycemic control, and less hypoglycemia and weight gain (in adults) compared with neutral protamine Hagedorn (NPH) insulin 16–19. Similarly, good glycemic control and a low risk of hypoglycemia have been reported from two randomized, controlled clinical trials carried out in children 20–21.

Results seen in randomized, controlled trials with IDet have been supported by those from observational studies. Observational studies are a valuable tool for investigating the efficacy and safety of therapeutics in a routine clinical setting. The PREDICTIVE (Predictable Results and Experience in Diabetes through Intensification and Control to Target: An International Variability Evaluation) study evaluated the safety and efficacy of IDet in clinical practice in over 40 000 adults globally (>25 countries) with T1D or type 2 diabetes (T2D) 22,23. Previously published results from cohorts of the PREDICTIVE study have shown that switching to IDet reduced major and nocturnal hypoglycemia and improved HbA1c in adult patients with T1D 23–24.

This analysis describes data from the Swedish pediatric and adolescent cohort of the PREDICTIVE study. In the main part, PREDICTIVE explored the effect of switching from NPH insulin or insulin glargine to IDet. In this current cohort, both recently diagnosed insulin-naïve patients and those with established diabetes (ED) who were switching basal insulin were included. The main objective was to evaluate the incidence of serious adverse drug reactions (SADRs), including major hypoglycemic events, following initiation of IDet in children and adolescents with T1D.

## Methods

### Study design

This was a multicenter, open-label, non-randomized, observational study investigating the safety of initiating IDet treatment in the Swedish pediatric and adolescent cohort of the PREDICTIVE study. This 26-wk study was carried out at 28 sites across Sweden between 2008 and 2010, with patients followed from initiation of treatment and at 12 and 26 wk thereafter. The primary objective was to observe the incidence of SADRs, including hypoglycemia, following initiation of IDet.

A detailed methodology for the PREDICTIVE study has been published 22. This study was approved by the research ethical committee in Lund, Sweden, and was carried out in accordance with the Declaration of Helsinki, good clinical practice, and local regulations. Written consent was obtained from the children’s parents or legal representative before any trial-related activity.

### Patients

A nonrandomized sample of approximately 200 children and adolescents aged between 6 and 18 yr was sought. Patients were enrolled at their physician’s discretion, as a result of routine clinical evaluation. Patients <18 yr of age who were IDet-naïve were eligible for inclusion. This includes patients who were insulin-naïve and those who were switching from another basal insulin. Patients were excluded if they were currently treated with IDet, had been diagnosed with T2D, or were known or suspected to be allergic to the trial product.

### Treatment

Children and adolescents were treated with IDet (Levemir; Novo Nordisk, Bagsværd, Denmark) (100 U/mL). Physicians determined the starting dose and injection frequency, as well as subsequent alterations to dose or injection frequency. Additionally, patients were treated with bolus insulin, either insulin lispro or insulin aspart, at their physician’s discretion.

### Study variables

Physicians obtained information from patient recall, the patient’s medical records, and the patient’s self-monitored plasma glucose (SMPG) diary. Information gathered at baseline included eligibility, demographic data, weight and height, disease duration, current insulin therapy, and number of overall, nocturnal, and major hypoglycemic episodes^1^ experienced over the past 4 wk (via patient recall), most recent HbA1c reading, and the six most recent fasting plasma glucose (FPG) readings.

Physicians also recorded the reason for starting IDet, starting dose, and any changes to concomitant therapy. Hypoglycemic and adverse events were captured from patient recall and the most recent HbA1c and FPG values were recorded.

### Safety measures

The primary outcome measure was the incidence of SADRs, including major hypoglycemia, following treatment with IDet. The incidence of all other adverse events was also recorded. Body weight was recorded at baseline, interim, and final visits.

### Efficacy measures

HbA1c, mean FPG, FPG variability, and insulin dose were recorded at baseline and at weeks 12 and 26. All HbA1c values were measured as Mono-S (%) and have been transformed to NGSP (%) and IFCC (mmol/mol) standards.^2^

### Statistical analysis

Data presented are for the observed cases (OC) study population, comprising all the patients who were observed at a defined time point. Continuous variables were summarized with descriptive statistics. Discrete variables were displayed in frequency tables. The primary outcome measure was summarized, with the number of events, the number and percentage of patients classified by system organ class, and by intensity and drug relation. Furthermore, as only one major SADR (major hypoglycemia) was reported, no modeling was done. Change in HbA1c and FPG from baseline were analyzed for the group with ED. No statistical analyses to assess efficacy were carried out in the newly diagnosed (ND) group because, following diagnosis, patients typically reduce HbA1c and FPG, and gain the weight they had lost prior to diagnosis with any insulin regimen. Furthermore, no comparisons between the ND and ED patients were planned or performed.

## Results

### Disposition of study patients

**Figure 1:**
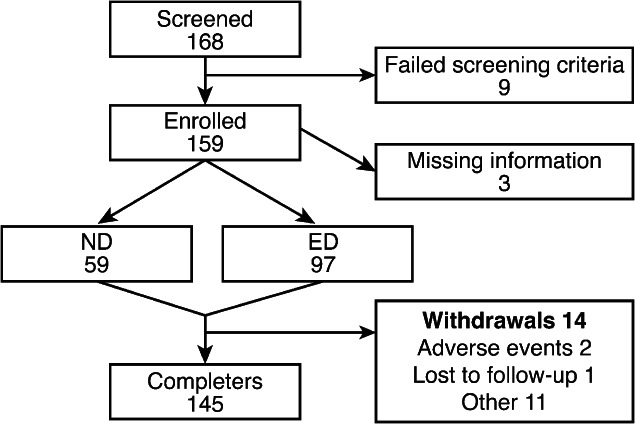
Subject disposition.

A total of 168 children and adolescents was enrolled in the study, of which 159 were eligible for inclusion in either the ND T1D (n = 59) or the ED (n = 97) stratum (data were missing for three patients so they were not included in the analysis) (Fig. 1). Of these, 145 completed the study; 14 were withdrawn due to adverse events, lost to follow-up or the withdrawal reason was not recorded. Within the ED stratum, 54% (n = 52) of patients switched from insulin glargine and 20% (n = 19) switched from NPH insulin. The remaining patients in the ED stratum had previously been treated with another insulin regimen, for example basal-only insulin pump therapy or twice-daily premixed insulin.

Baseline characteristics of the ND and ED groups are summarized in Table 1. The mean age was 9.7 yr in the ND stratum (range 0–15 yr), and 12.5 yr in the ED stratum (range 2–18 yr). The BMI for both strata were within three standard deviations (SD) of the reference population mean, according to Karlberg’s classification for Swedish children 25. The mean disease duration was 4 yr for the ED stratum. The majority of children in the ED group used a basal–bolus insulin regimen prior to the start of the study.

**Table 1:** Baseline characteristics

Characteristics	Newly diagnosed T1D (n = 59)	Established T1D (n = 97)
Female/male* (%)	37.9/62.1	52.6/47.4
Age (yr)	9.7 (3.8)	12.5 (3.2)
Height (cm)	146.9 (20.8)	157.8 (18.0)
Weight (kg)	40.0 (19.1)	53.9 (20.1)
BMI (kg/m^2^)	18.2 (4.3)	21.0 (4.4)
Diabetes duration (yr)	0 (0)	3.9 (3.8)
HbA1c		
%	11.1 (1.9)	8.2 (1.6)
mmol/mol	97.2 (21.1)	66.3 (17.5)
FPG (mmol/L)	7.6 (2.8)	9.1 (3.9)

BMI, body mass index, FPG, fasting plasma glucose; SD, standard deviation; T1D, type 1 diabetes.

Values are mean (SD) unless otherwise stated.

* Four patients had missing data on gender.

In the ED stratum, 61.9% of patients had experienced at least one hypoglycemic episode in the 4 wk prior to baseline. Patients in the ND group had typically been treated with intravenous insulin usually for 2–3 d following diagnosis with T1D and prior to the initiation of IDet as part of a basal–bolus insulin regimen; 19.3% had experienced a hypoglycemic event during this period.

The most common reasons given by physicians for starting treatment with IDet were: initiating insulin therapy (50%), to improve glycemic control (24.4%), to reduce PG variability (19.9%), patient dissatisfaction with current therapy (17.3%), and to improve weight control (12.8%).

### Adverse events

The primary endpoint of this study was the occurrence of SADRs. Only one patient, in the ND stratum, reported a major hypoglycemic event occurring in the 4 wk preceding the final visit, which was classified as serious and as a result the insulin dose was reduced.

In one patient in the ED stratum, IDet was withdrawn due to injection-site reactions. All other adverse events were classified as mild.

### Hypoglycemia

**Figure 2:**
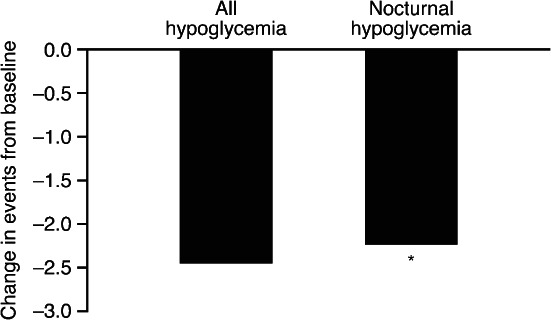
Change in mean number of nocturnal hypoglycemic events in the ED stratum. Change (baseline to 26 wk) in mean number of events in the 4 wk prior to data collection for established diabetes (ED) stratum. *p < 0.05.

The number of hypoglycemic events reported by both strata are summarized in Table 2. Five patients in the ED stratum had experienced a major hypoglycemic episode in the 4 wk prior to baseline; none of the patients in this stratum reported major hypoglycemia during the study. There was a trend toward a reduction in mean number of hypoglycemic events from 8.97 in the 4 wk prior to baseline to 7.25 in the 4 wk prior to the final visit (mean reduction of 2.46 events, p = 0.0622, not significant) in the ED stratum (Fig. 2). There was a significant reduction in the occurrence of nocturnal hypoglycemia from baseline to final visit in the ED stratum, with a mean reduction of 2.24 events, p = 0.0078 (Fig. 2).

**Table 2:** Summary of hypoglycemic events in the 4 wk leading up to a visit

	Newly diagnosed T1D (n = 14)*	Established T1D (n = 62)*
Overall hypoglycemic events		
Baseline	—	8.97 (10.95)
Final visit	7.36 (6.99)	7.25 (8.40)
Day-time hypoglycemic events		
Baseline	—	7.43 (8.53)
Final visit	6.82 (6.98)	6.88 (7.88)
Nocturnal hypoglycemic events		
Baseline	—	1.80 (4.02)
Final visit	0.55 (1.14)	0.38 (1.07)

SD, standard deviation; T1D, type 1 diabetes.

Values are mean (SD) events occurring in the 4 wk prior to a visit, based on patient recall. Baseline data are not presented for the newly diagnosed cohort because they were treated with intravenous insulin rather than a basal insulin prior to this study.

* Data were missing for some patients; only the observed cases are included in this analysis.

At final visit, there was a mean of 7.36 overall hypoglycemic and 0.55 nocturnal hypoglycemic events occurring in the ND stratum (Table 2). One major hypoglycemic event was reported in the ND stratum during the study.

### Glycemic control

**Figure 3:**
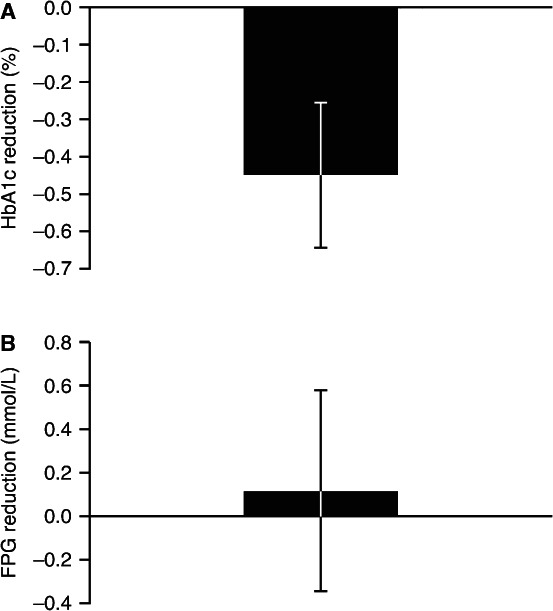
Change from baseline in (A) HbA1c and (B) fasting plasma glucose after 26 wk’ treatment with IDet in the ED stratum. Data are mean ± SEM. Change is for the observed cases (OC) population in the established diabetes (ED) stratum. FPG, fasting plasma glucose; IDet, insulin detemir.

At baseline, patients in the ND stratum had a mean HbA1c of 11.05 ± 1.9% (97.2 ± 21.1 mmol/mol), which decreased to 6.8 ± 1.0% (51.1 ± 11.0 mmol/mol) after 26 wk of treatment. In the ED stratum, mean (±SD) HbA1c decreased from 8.2 ± 1.6% (66.3 ± 17.5 mmol/mol) at baseline to 7.7 ± 1.2% (60.8 ± 12.8 mmol/mol) at final visit (Fig. 3A); the mean reduction of −0.45% (−4.89 mmol/mol) was not statistically significant, p = 0.16. At baseline 35.0% of patients in the ED stratum were at HbA1c target <7.5% (58 mmol/mol); at the end of study this had increased to 46.3% of patients.

Mean FPG decreased from 7.6 ± 2.8 mmol/L at baseline to 7.2 ± 2.3 mmol/L at final visit in the ND stratum, and from 9.1 ± 3.9 to 8.6 ± 3.0 mmol/L after 26 wk in the ED stratum (Fig. 3B). There was no significant change in FPG over time in the ED stratum. Additionally, the variability in FPG was calculated. Mean variability of FPG (CV, %) was 27.9% at baseline and 22.8% at final visit for the ND stratum. For the ED stratum, variability in FPG was 33.9 and 30.3% at baseline and final visits, respectively. There was no significant difference between baseline and final visit for the ED stratum (p = 0.17).

### Insulin dose

All patients followed a basal–bolus regimen during the study. Basal insulin doses are summarized in Table 3. At baseline, ND patients were started on 0.53 ± 0.22 U/kg IDet and those with ED on 0.45 ± 0.18 U/kg IDet. At the final visit, the total daily dose of IDet was 0.39 ± 0.18 U/kg and 0.54 ± 0.20 U/kg for ND and ED strata, respectively. Patients in the ND stratum started on a mean total insulin dose of 1.13 ± 0.50 U/kg and decreased to 0.74 ± 0.30 U/kg at final visit. After 26 wk, the total insulin dose had significantly increased from 0.88 ± 0.39 to 0.98 ± 0.31 U/kg in the ED stratum (p < 0.0001). At baseline, 23% of patients in the ED stratum received IDet once daily and 77% administered it twice daily. At the end of the study, 16 and 84% administered IDet once or twice daily, respectively. The decision to dose IDet once or twice daily reflected the normal clinical practice at the particular centers.

**Table 3:** Basal insulin dose

Visit	Newly diagnosed T1D (n = 59)	Established T1D (n = 97)
Pre-dose		0.45 (0.18)
Baseline – week 0	0.53 (0.22)	0.45 (0.20)
Interim – week 12	0.34 (0.16)	0.51 (0.20)
Final visit – week 26	0.39 (0.18)	0.54 (0.20)

SD, standard deviation; T1D, type 1 diabetes.

Values are mean (SD) in insulin U/kg.

### Weight

**Figure 4:**
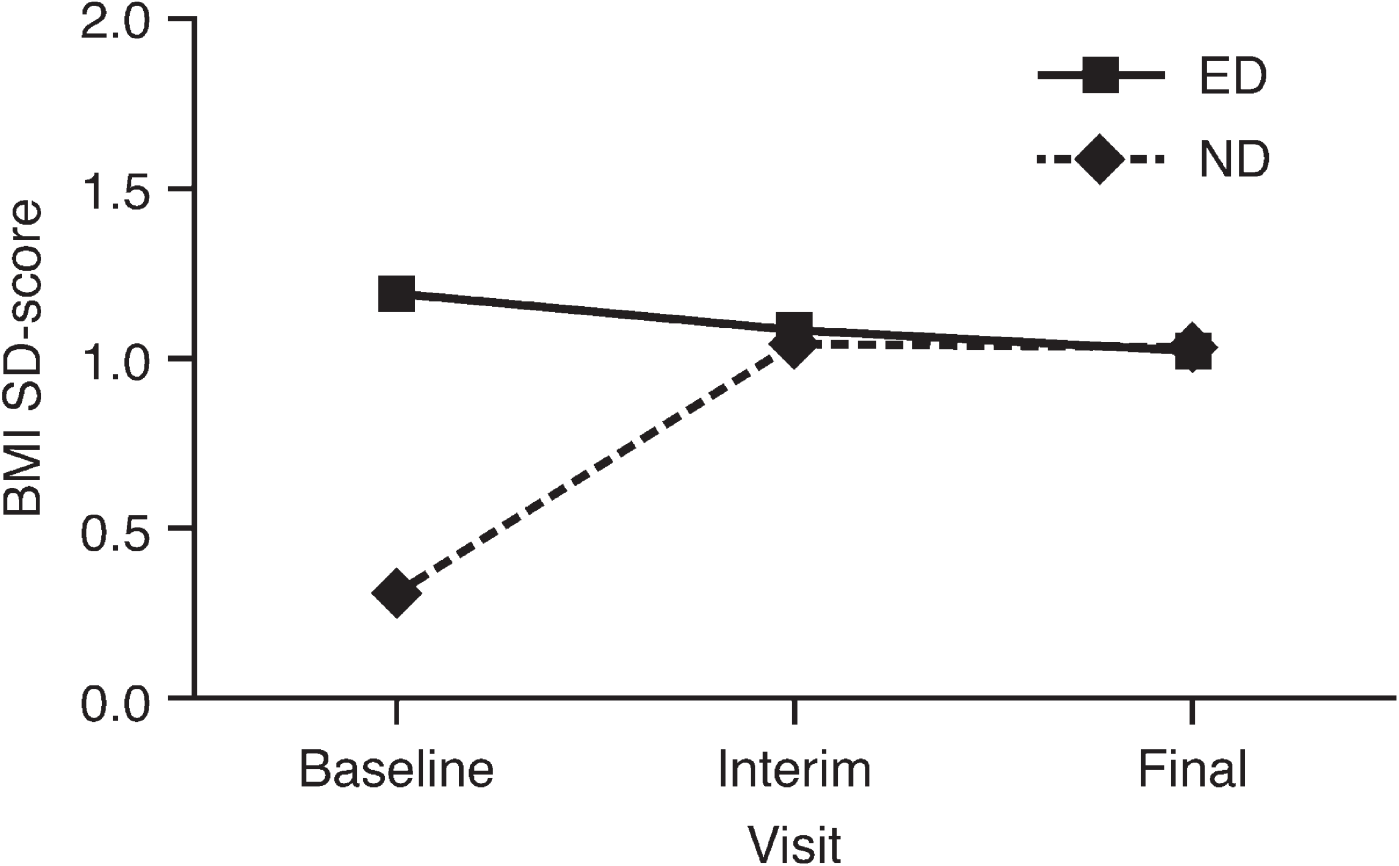
Change in standardized body mass index (BMI). ED, established diabetes; ND, newly diagnosed; SD, standard deviation.

Mean weight increased by 5.7 kg in the ND stratum and 2.0 kg in the ED stratum from baseline to study end (p < 0.0001). The weight increase of the ED stratum was comparable to that of the reference population. At baseline, 96% of patients in both the ND and ED strata had a BMI within the limits (±3 SD) of the reference population 25, respectively, and at final visit, 94% in the ND stratum and 98% in the ED stratum were within the limits. The change in BMI from baseline to final visit (0.2 kg/m^2^) in the ED stratum was not significant. The mean BMI–SD score for the ED stratum, which takes the patient’s height increase into consideration and compares with a reference population 25, did not increase during the study (Fig. 4). The mean BMI–SD score increased from baseline to the interim visit (week 12) in the ND stratum, but did not increase thereafter (Fig. 4).

## Discussion

This observational study in a diverse cohort of 159 children and adolescents (age range 0–18 yr) with T1D shows that IDet was well tolerated in both insulin-naïve children and those switching to IDet from another basal insulin. IDet provided good glycemic control, a reduced risk of nocturnal confirmed hypoglycemia, and no inappropriate weight gain. The main outcome measure here was the incidence of SADRs; only one event that was classified as serious occurred during the study, so no statistical analysis could be performed. The low frequency of SADRs in this study might indicate that IDet is well tolerated in young patients, which is supported by findings from another PREDICTIVE youth cohort in which there were no SADRs 26. It has been shown that fear of severe hypoglycemia is particularly apparent among parents of children with diabetes 13; therefore, the low rate of hypoglycemia with IDet in children is particularly relevant.

The EU label for IDet has recently been updated to include an indication for treatment of children aged 2 yrs and above. Owing to the observational nature of this study, 12 of the included patients were under the age of 6 yr at the start of this study (nine and three patients in the ND and ED strata, respectively) and are in line with a previous study 21 illustrating that IDet is a suitable basal insulin for young children (aged 2 yrs and upwards).

After 26 wk, patients in the ND stratum had improved glycemic control; an improvement of this magnitude can be expected in ND patients with T1D. Small but nonsignificant decreases in HbA1c and FPG were observed in the ED stratum from baseline, suggesting that IDet provided similar glycemic control to other basal insulins. No statistical analysis was conducted between the ND and ED groups, as they are not comparable. The results of this study were in accordance with the Turkish PREDICTIVE youth cohort of 101 patients aged 6–17 yr, in which switching to IDet significantly improved HbA1c (−0.7%, compared to a nonsignificant reduction of −0.45% in the ED stratum in this study) 26. The lack of significance in this study may be due to the smaller cohort of patients switching from another basal insulin to IDet. The American Diabetes Association recommends HbA1c targets of between 7.5 and 8.5% for children under 6 yr of age, of ≤8% for children aged 6–12 yr, and of <7.5% for adolescents (aged 13–19 yr) 27, and the ISPAD guidelines recommend a target of <7.5% in children and adolescents 28. Therefore, the end-of-study HbA1c of 6.8% for the ND stratum (mean age of 9.7 yr) and 7.7% for the ED stratum (with a mean age 12.5 yr) indicates that many children reached these targets (46.3% patients in the ED stratum with HbA1c <7.5% at end of study). Results from the adult PREDICTIVE cohorts demonstrated improved glycemic control following a switch to IDet, but these studies included much larger numbers of patients 23–24. Randomized, controlled trials have shown both comparable 19 and superior 16 glycemic control with IDet compared with NPH insulin.

Mean basal insulin doses significantly increased from baseline to end of study in the ED stratum. This may, in part, be because physicians cited ‘to improve glycemic control’ as a major reason for initiating IDet therapy. Alternatively, it could be because many patients switched from once-daily insulin glargine to twice-daily IDet, and higher doses of basal insulin are sometimes observed with twice-daily compared with once-daily basal insulin dosing 29. The increase in insulin dose during the study was coupled with a numerically lower HbA1c at the end of study. A previous controlled trial in children showed that end-of-trial doses of IDet were similar to NPH insulin, as were end-of-trial HbA1c levels 21–30. Additionally, higher basal doses may have been required because patients underestimated their bolus doses.

Importantly, switching basal insulin to IDet resulted in a numerical reduction in overall and day-time hypoglycemic events, and significantly fewer nocturnal hypoglycemic events. In the Turkish PREDICTIVE youth study, both overall and nocturnal hypoglycemia were significantly reduced from baseline after 12 wk’ treatment with IDet. A reduction in nocturnal hypoglycemia in children treated with IDet compared to NPH has also been shown in controlled trials 20–21, which are more reliable. The data in this study are also in agreement with the results from the adult PREDICTIVE cohort 23–24. Hypoglycemia is often reported as the major limiting factor in achieving good glycemic control in diabetes 31. The reduction in incidence of hypoglycemia, in particular nocturnal hypoglycemia, seen here, and in other studies with IDet, suggests that more aggressive titration of insulin dose may be possible to achieve better glycemic control and reduce the development of future macro-and microvascular complications. However, it should be noted that, as some of the patients in this study switched to IDet to reduce hypoglycemia, it is possible that any change of treatment may have caused a reduction in hypoglycemia, or that natural fluctuations in the disease course may have resulted in an observed decrease in hypoglycemia.

In addition to hypoglycemia, inappropriate weight gain following treatment with insulin is a major barrier to achieving glycemic control in teenagers and adults with T1D. Results from randomized, controlled trials have shown that IDet results in less weight gain than NPH insulin 16–19, and this has also been reported in children 20–21. In this study, BMI was classified as within 3 SD of the reference population mean 25, suggesting that IDet did not cause inappropriate weight gain in children and adolescents. The moderate increase in BMI in the ED stratum was expected, as the BMI of a growing child increases by approximately this amount with increasing age 25 and the BMI-SD score did not increase during this study. The weight increase observed in the ND stratum was also expected, because children typically lose weight prior to diagnosis with T1D.

Observational studies provide an important and necessary supplement to controlled trials, contributing valuable information on the use, safety, and efficacy of therapies in clinical practice. However, observational studies rely on patient recall for the reporting of hypoglycemic events, so it must be acknowledged that this recall may not be completely accurate. Owing to the low patient numbers in this cohort, it is possible that the frequency of some adverse events may have been missed or under-represented. Furthermore, any change of regimen may have positive effects on metabolic control due to an increased interest in self-management by the patients and their carers. Moreover, it is possible that some of the improvements observed are due to a bias in patient selection. As this was an observational study, there was no control group, making it difficult to ascertain whether the outcomes were directly related to the study medication. However, as there are relatively few controlled trials examining the effects of IDet in children and adolescents 20,21, this observational study provides important data on its efficacy and safety in young patients in clinical practice.

## Conclusion

Overall, the results of this observational study support those seen in controlled clinical trials in children and adolescents, as well as those in adult patients: IDet is a well-tolerated and efficacious basal insulin. IDet is suitable for use in ND children and adolescents, and those wanting to switch basal insulin to reduce the risk of hypoglycemia, especially nocturnal hypoglycemia.

## Footnotes

1 Defined as blood glucose <2.8 mmol/L or symptomatic hypoglycemic resolved with oral carbohydrate intake or intravenous glucose. Nocturnal hypoglycemia was defined as occurring after the evening insulin injection and before morning determination of FPG and morning insulin injection. Major hypoglycemic events were defined as those requiring the assistance of another person.

2 http://www.diabeteschart.org/ptom.html

## References

[b1] Nordwall M, Arnqvist HJ, Bojestig M, Ludvigsson J (2009). Good glycemic control remains crucial in prevention of late diabetic complications – the Linkoping Diabetes Complications Study. Pediatr Diabetes.

[b2] DCCT Research Group (1993). The effect of intensive treatment of diabetes on the development and progression of long-term complications in insulin-dependent diabetes mellitus. N Engl J Med.

[b3] DCCT Research Group (1994). Effect of intensive diabetes treatment on the development and progression of long-term complications in adolescents with insulin-dependent diabetes mellitus: Diabetes Control and Complications Trial. J Pediatr.

[b4] Bojestig M, Arnqvist HJ, Hermansson G, Karlberg BE, Ludvigsson J (1994). Declining incidence of nephropathy in insulin-dependent diabetes mellitus. N Engl J Med.

[b5] Nordwall M, Bojestig M, Arnqvist HJ, Ludvigsson J (2004). Linköping Diabetes Complications Study. Declining incidence of severe retinopathy and persisting decrease of nephopathy in an unselected population of type 1 diabetes – the Linkoping Diabetes Complications Study. Diabetologia.

[b6] UK Prospective Diabetes Study (UKPDS) Group (1998). Intensive blood-glucose control with sulphonylureas or insulin compared with conventional treatment and risk of complications in patients with type 2 diabetes (UKPDS). Lancet.

[b7] Davis EA, Keating B, Byrne GC, Russell M, Jones TW (1997). Hypoglycemia: incidence and clinical predictors in a large population-based sample of children and adolescents with IDDM. Diabetes Care.

[b8] Bjørgaas MR (2012). Cerebral effects of severe hypoglycaemia in young people with type 1 diabetes. Pediatr Diabetes.

[b9] Ho MS, Weller NJ, Ives FJ (2008). Prevalence of structural central nervous system abnormalities in early-onset type 1 diabetes mellitus. J Pediatr.

[b10] Lunetta M, Damanti AR, Fabbri G, Lombardo M, Di Mauro M, Mughini L (1994). Evidence by magnetic resonance imaging of cerebral alterations of atrophy type in young insulin-dependent diabetic patients. J Endocrinol Invest.

[b11] Perros P, Deary IJ, Sellar RJ, Best JJ, Frier BM (1997). Brain abnormalities demonstrated by magnetic resonance imaging in adult IDDM patients with and without a history of recurrent severe hypoglycemia. Diabetes Care.

[b12] Ryan C, Gurtunca N, Becker D (2005). Hypoglycemia: a complication of diabetes therapy in children. Pediatr Clin North Am.

[b13] Barnard K, Thomas S, Royle P, Noyes K, Waugh N (2010). Fear of hypoglycaemia in parents of young children with type 1 diabetes: a systematic review. BMC Pediatr.

[b14] Davis NL, Bursell JD, Evans WD, Warner JT, Gregory JW (2012). Body composition in children with type 1 diabetes in the first year after diagnosis: relationship to glycaemic control and cardiovascular risk. Arch Dis Child.

[b15] Hanberger L, Samuelsson U, Berterö C, Ludvigsson J (2012). The influence of structure, process, and policy on HbA1c levels in treatment of children and adolescents with type 1 diabetes. Diabetes Res Clin Pract.

[b16] Bartley PC, Bogoev M, Larsen J, Philotheou A (2008). Long-term efficacy and safety of insulin detemir compared to neutral protamine Hagedorn insulin in patients with type 1 diabetes using a treat-to-target basal–bolus regimen with insulin aspart at meals: a 2-year, randomized, controlled trial. Diabet Med.

[b17] Russell-Jones D, Bolinder J, Simpson R (2002). Lower and more predictable fasting blood glucose and reduced risk of nocturnal hypoglycaemia with once daily insulin detemir versus NPH in subjects with type 1 diabetes. Diabetologia.

[b18] Haak T, Tiengo A, Draeger E, Suntum M, Waldhäusl W (2005). Lower within-subject variability of fasting blood glucose and reduced weight gain with insulin detemir compared to NPH insulin in patients with type 2 diabetes. Diabetes Obes Metab.

[b19] Vague P, Selam JL, Skeie S (2003). Insulin detemir is associated with more predictable glycemic control and reduced risk of hypoglycemia than NPH insulin in patients with type 1 diabetes on a basal-bolus regimen with premeal insulin aspart. Diabetes Care.

[b20] Robertson KJ, Schoenle E, Gucev Z, Mordhorst L, Gall MA, Ludvigsson J (2007). Insulin detemir compared with NPH insulin in children and adolescents with type 1 diabetes. Diabet Med.

[b21] Thalange N, Bereket A, Larsen J, Hiort LC, Peterkova V (2011). Treatment with insulin detemir or NPH insulin in children aged 2–5 yr with type 1 diabetes mellitus. Pediatr Diabetes.

[b22] Lüddeke HJ, Sreenan S, Aczel S, PREDICTIVE Study Group (2007). PREDICTIVE™ – a global, prospective observational study to evaluate insulin detemir treatment in types 1 and 2 diabetes: baseline characteristics and predictors of hypoglycaemia from the European cohort. Diabetes Obes Metab.

[b23] Sreenan S, Virkamäki A, Zhang K, Hansen JB (2008). PREDICTIVE study group. Switching from NPH insulin to once-daily insulin detemir in basal–bolus-treated patients with diabetes mellitus: data from the European cohort of the PREDICTIVE™ study. Int J Clin Pract.

[b24] Dornhorst A, Lüddeke HJ, Honka M, PREDICTIVE Study Group (2008). Safety and efficacy of insulin detemir basal-bolus therapy in type 1 diabetes patients: 14-week data from the European cohort of the PREDICTIVE study. Curr Med Res Opin.

[b25] Karlberg J, Luo ZC, Albertsson-Wikland K (2001). Body mass index reference values (mean and SD) for Swedish children. Acta Paediatr.

[b26] Kurtoglu S, Atabek ME, Dizdarer C (2009). Insulin detemir improves glycemic control and reduces hypoglycemia in children with type 1 diabetes: findings from the Turkish cohort of the PREDICTIVE™ observational study. Pediatr Diabetes.

[b27] Silverstein J, Klingensmith G, Copeland K, American Diabetes Association (2005). Care of children and adolescents with type 1 diabetes: a statement of the American Diabetes Association. Diabetes Care.

[b28] Global IDF/ISPAD guideline for diabetes in childhood and adolescence (2011). http://www.ispad.org/NewsFiles/IDF-ISPAD_Diabetes_in_Childhood_and%20Adolescence_Guidelines_2011.pdf.

[b29] Rosenstock J, Davies M, Home PD, Jarsen J (2008). A randomised, 52-week, treat-to-target trial comparing insulin detemir with insulin glargine when administered as an add-on to glucose-lowering drugs in insulin-naïve people with type 2 diabetes. Diabetologia.

[b30] Thalange N, Bereket A, Larsen J, Hiort LC, Peterkova V (2013). Insulin analogues in children with type 1 diabetes: a 52-week randomized clinical trial. Diabet Med.

[b31] Cryer PE (2002). Hypoglycemia: the limiting factor in the glycaemic management of type 1 and type II diabetes. Diabetologia.

